# A multicentre randomised controlled trial assessing whether MRI-targeted biopsy is non-inferior to standard transrectal ultrasound guided biopsy for the diagnosis of clinically significant prostate cancer in men without prior biopsy: a study protocol

**DOI:** 10.1136/bmjopen-2017-017863

**Published:** 2017-10-12

**Authors:** Veeru Kasivisvanathan, Fatima Jichi, Laurence Klotz, Arnauld Villers, Samir S Taneja, Shonit Punwani, Alex Freeman, Mark Emberton, Caroline M Moore

**Affiliations:** 1 Division of Surgery and Interventional Science, University College London, London, UK; 2 Department of Urology, University College London Hospital, London, UK; 3 Biostatistics Group, Joint Research Office, University College London and University College London Hospital, London, UK; 4 Department of Urology, Sunnybrook Hospital, Toronto, Canada; 5 Department of Urology, CHU Lille, University Lille Nord de France, Lille, France; 6 Department of Urology, New York University Langone Medical Centre, New York City, New York, USA; 7 Centre for Medical Imaging, University College London, London, UK; 8 Department of Radiology, University College London Hospital, London, UK; 9 Department of Pathology, University College London Hospital, London, UK

**Keywords:** magnetic resonance imaging, prostate disease, urology, biopsy, diagnosis, randomised

## Abstract

**Introduction:**

The classical pathway for the diagnosis of prostate cancer is transrectal ultrasound-guided (TRUS) biopsy of the prostate initiated on the basis of a raised prostate-specific antigen (PSA). An alternative pathway is to perform multi-parametricMRI (MPMRI) to localise cancer and to use this information to influence the decision for, and conduct of, a subsequent biopsy, known as an MPMRI-targeted biopsy. An MPMRI pathway has been shown to detect a similar or greater amount of clinically significant cancer as TRUS biopsy but has several advantages, including the potential to biopsy fewer men with fewer cores.

**Methods:**

This is a pragmatic, international, multicentre, parallel group randomised study in which men are allocated in a 1:1 ratio to an MPMRI or TRUS biopsy pathway. This study will assess whether an MPMRI-targeted biopsy approach is non-inferior to a standard TRUS biopsy approach in the diagnosis of clinically significant cancer.

Men in the MRI arm will undergo targeted biopsy of suspicious areas only and no biopsy will be carried out if the MRI is non-suspicious. Men in the TRUS biopsy will undergo a standard 10–12-core TRUS biopsy. The main inclusion criteria are a serum PSA ≤20 ng/mL, a digital rectal examination finding of T2 or less and no prior prostate biopsy.

The primary outcome is the proportion of men with clinically significant cancer detected. A sample size of at least 470 patients is required. Key secondary outcomes include the proportion of clinically insignificant cancer detected.

**Ethics and dissemination:**

Ethical approval was obtained from the National Research Ethics Committee East Midlands, Leicester (15/EM/0188). Results of this study will be disseminated through national and international papers. The participants and relevant patient support groups will be informed about the results of the study.

**Registration details:**

NCT02380027; Pre-results

**Strengths and limitations of this study**

- Randomised trial which mitigates bias and reduces chances of observed outcome being influenced by confounding factors.
- International multicentre study making results more generalisable.
- Pragmatic design making results more applicable to real life clinical practice, thus results can be used to change clinical practice.
- Pragmatic design introducing more variability in trial interventions.

## Study title

Long study title: A multicentre randomised controlled trial assessing whether magnetic resonance imaging-targeted biopsy is non-inferior to standard transrectal ultrasound guided biopsy for the diagnosis of clinically significant prostate cancer in men without prior biopsy

Short study title: PRostate Evaluation for Clinically Important disease: Sampling using Image-guidance Or Not? (PRECISION)

Study Acronym: PRECISION

## Introduction

Prostate cancer is the most common male cancer in Europe with an incidence of 370 000 new cases per year and an incidence in the USA of 161 360 new cases per year.^1 2^ It is the second most common cause of cancer death in European men, with 90 000 deaths per year in Europe and 26 730 deaths per year in the USA.^1 2^ A major unmet need in prostate cancer care is to better identify men who will benefit from treatment while avoiding overtreatment of men who are unlikely to benefit.

The classical pathway for the diagnosis of prostate cancer is transrectal ultrasound-guided (TRUS) biopsy of the prostate following a raised prostate-specific antigen (PSA). TRUS guidance is performed primarily for anatomical guidance and the ultrasound discriminates poorly between cancerous and non-cancerous tissues.^3^ Biopsies are concentrated in areas of the peripheral zone, thought to harbour the majority of cancers.^4^

An alternative pathway for the diagnosis of prostate cancer in men with raised PSA is to perform a multi-parametric MRI (MPMRI) to localise cancer and to use this information to influence both the decision for and conduct of a subsequent biopsy, known as an MPMRI-targeted biopsy. MPMRI has demonstrated good diagnostic performance for the detection of clinically significant cancer.^5^ MPMRI-targeted biopsy has been shown to detect a similar or greater amount of clinically significant cancer to TRUS biopsy but avoid the detection of a greater proportion of clinically insignificant cancer, which may not benefit from treatment.^6^ Robust level 1 evidence from appropriately powered studies comparing MPMRI-targeted biopsy to TRUS biopsy in a pragmatic multicentre setting is lacking.

If MPMRI-targeted biopsy leads to fewer men being biopsied with fewer biopsy cores, no fewer clinically significant cancers identified but fewer insignificant cancers identified, then an MPMRI-driven diagnostic pathway could replace TRUS biopsy as the standard of care for prostate cancer diagnosis.

### Objectives

The primary objective is to assess whether MPMRI-targeted biopsy is non-inferior to standard 10–12-core TRUS biopsy in the diagnosis of clinically significant prostate cancer in men referred with clinical suspicion of prostate cancer who have had no prior prostate biopsy.

Key secondary objectives include:

- To assess whether MPMRI-targeted biopsy detects fewer men with clinically insignificant prostate cancer than 10–12-core TRUS biopsy
- To assess the proportion of men in the MRI arm who avoid biopsy

### Trial design

**Figure 1:**
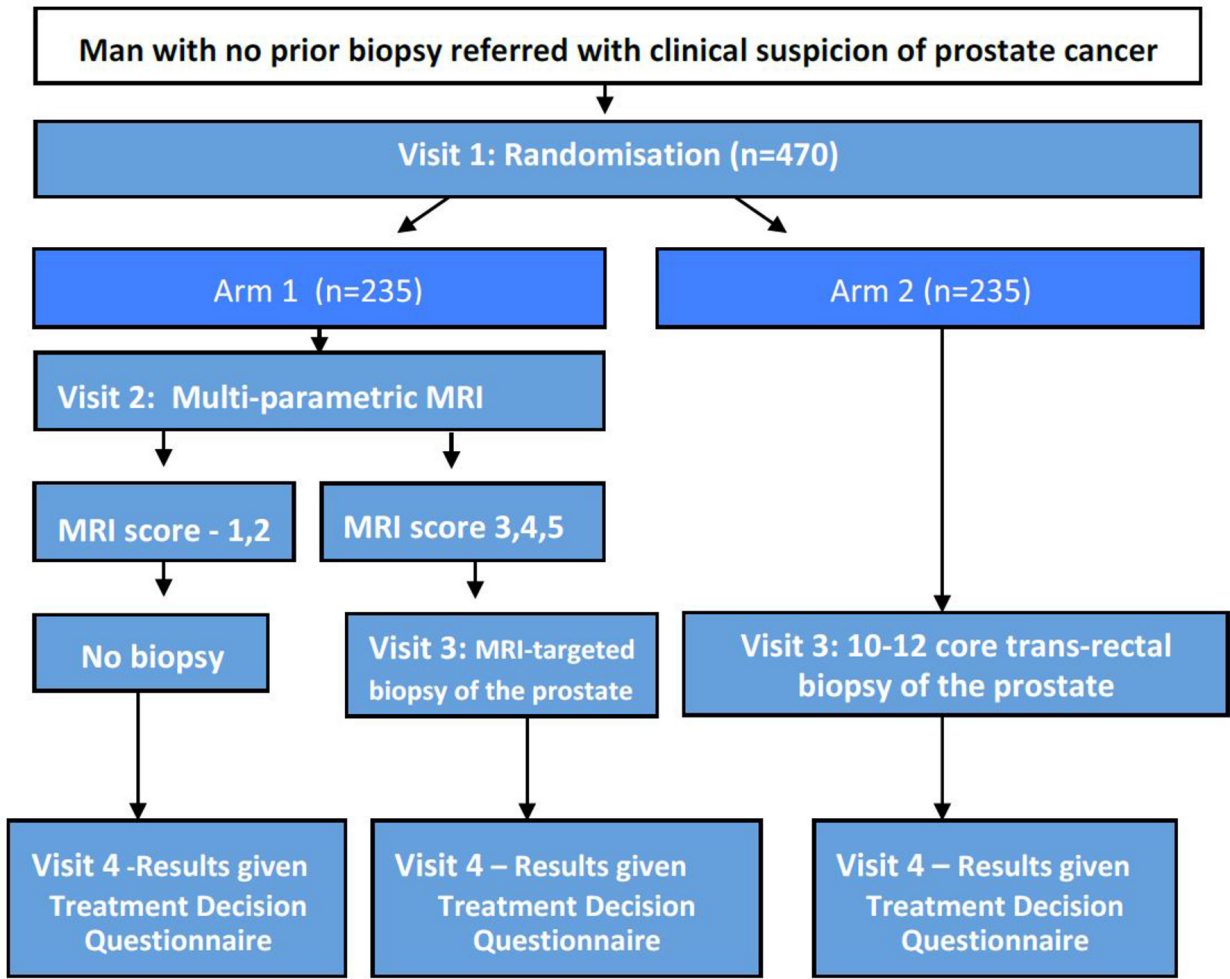
PRECISION Trial schema.

This is a pragmatic, international, multicentre, parallel group, non-inferiority randomised study in which men are allocated in a 1:1 ratio to MPMRI or to TRUS biopsy. This is the first trial to randomise men to an MRI arm in which targeted biopsy alone is carried out in the presence of an MRI lesion (ie, no biopsies of MRI-normal areas of prostate) and in which no biopsy at all is carried out if the MRI is non-suspicious (figure 1). The comparator of TRUS biopsy has been chosen as this is currently the standard of care in the majority of institutions.

A randomised trial design was chosen rather than a paired cohort design for the following reasons:

- In order to reduce performance bias that can result from carrying out one test after another as the conduct of one test may influence conduct of the other when both are carried out in the same patient.
- To allow the assessment of adverse events (AEs) associated with each technique.
- To allow the assessment of the patient’s willingness to remain on their allocated pathway.
- To allow the assessment of the physician’s willingness to adhere to the allocated pathway.
- To allow patients to potentially benefit by having a biopsy with fewer biopsy cores.
- To allow patients to potentially benefit by avoiding a biopsy and its complications altogether.

There is no gold standard reference test that is applicable to all patients referred with suspected prostate cancer. As radical prostatectomy is only performed in a small proportion of patients who test positive on the index test, it is therefore not an appropriate reference test. Therefore, a comparison of cancer detection between each approach was chosen as the primary outcome of interest.

## Methods and analysis

### Study setting

Hospitals which perform MPMRI-targeted and TRUS biopsy will take part in the study. Some of these centres will be from the Standards of Reporting for MRI-targeted Biopsy Studies (START) consortium,^7^ though non-START consortium centres are permitted to take part providing prior detection rates from the biopsy procedures in their practice are approved by the chief investigator prior to starting the study at their centre.

We expect centres from the following countries to take part: Argentina, Belgium, Canada, Finland, France, Italy, Germany, the Netherlands, Switzerland, UK and the USA.

### Eligibility criteria

Patients will be considered eligible for registration into this study if they fulfil all of the inclusion criteria and none of the exclusion criteria, as defined in box 1. The key eligibility criteria include serum PSA ≤20 ng/mL, suspected tumour stage ≤T2 on rectal examination and no prior prostate biopsy.

Box 1 **Eligibility criteria**

**Inclusion criteria**

1. Men at least 18 years of age referred with clinical suspicion of prostate cancer who have been advised to have a prostate biopsy
2. Serum prostate-specific antigen (PSA) ≤20 ng/mL
3. Suspected stage ≤T2 on rectal examination (organ-confined prostate cancer)
4. Fit to undergo all procedures listed in the protocol
5. Able to provide written informed consent

**Exclusion criteria**

1. Prior prostate biopsy
2. Prior treatment for prostate cancer
3. Contraindication to MRI (eg, claustrophobia, pacemaker, estimated glomerular filtration rate ≤50 mls/min)
4. Contraindication to prostate biopsy
5. Men in whom artefact would reduce the quality of the MRI
6. Previous hip replacement surgery, metallic hip replacement or extensive pelvic orthopaedic metal work
7. Unfit to undergo any procedures listed in the protocol

### Interventions

#### MPMRI arm

MPMRI will be carried out using a 1.5 Tesla or 3.0 Tesla scanner with a pelvic phased array coil and power injector for contrast administration with the patient in the supine position. T2-weighted, diffusion weighted and dynamic contrast enhanced scans will be acquired as per the minimum requirements set out by consensus guidelines.^8^ The site performing the MPMRI should provide specific detailed information on their MRI scanners and protocols in advance which need to be approved by the PRECISION Operations Group prior to participating in the study.

The MPMRI will be reported by an experienced nominated radiologist using a dedicated MRI reporting pro forma. Prior experience of the radiologists must be provided in advance and approved by the PRECISION Operations Group prior to the site being able to participate in the study. The MPMRI will be scored based on consensus recommendations^8 9^ employing a 1–5 score of suspicion:

Suspicious areas in the prostate will be scored on the following scale:

- 1 = Highly unlikely to be clinically significant cancer
- 2 = Unlikely to be clinically significant cancer
- 3 = The presence of clinically significant cancer is equivocal
- 4 = Likely to be clinically significant cancer
- 5 = Highly likely to be clinically significant cancer

#### MPMRI-targeted biopsy

A participant with a prostate that contains a suspicious area with an MPMRI score of 3, 4 or 5 will subsequently undergo targeted biopsy by a clinician experienced in MPMRI-targeted biopsy. The experience of the clinicians performing biopsy must be provided to the PRECISION Operations Group in advance and approved prior to the site participating in the study.

A maximum of three MRI-suspicious areas will be chosen for targeted biopsy with a maximum of 4 cores to be taken per suspicious area and a maximum of 12 cores in total. Targeted biopsies can be performed by visual registration or software-assisted registration, as described previously,^7^ and can be carried out transrectally or transperineally, according to local expertise.

#### No target identified on MPMRI

A participant with a prostate that contains no suspicious areas on MPMRI (ie, scoring 1 or 2) will not undergo a biopsy as part of the protocol.

#### TRUS biopsy arm

All participants in this arm will undergo a standard 10–12-core TRUS biopsy as described previously.^10^ The patient will be positioned in the left lateral position and 10–12 cores will be taken from the peripheral zone of the apex, mid-gland and base of the prostate on the right and left sides.

The experience of the clinicians performing the biopsy must be provided to the PRECISION Operations Group in advance and approved prior to the site participating in the study.

#### Patient-reported outcome measures

In both arms self-reported questionnaires to capture biopsy-specific side effects will be completed immediately postprocedure, and at 30 days. Health-related quality of life EQ-5D-5L questionnaires will also be completed at baseline, 24 hours post-MRI, 24 hours post biopsy and 30 days post intervention.

#### Treatment decision and participant timeline

A clinician reviews participants with results of the test and treatment decisions are made according to local standard of care. If a further biopsy, further MPMRI or any other diagnostic test is requested from the treatment decision visit or if the clinician books a radical prostatectomy, then the result of the procedure is recorded, after which the man completes the study. Otherwise men complete the study after the treatment decision visit or when the 30-day questionnaires are completed, whichever is later.

Providing the interventions occur in the specified sequence order, there is no necessitated timeline within which the interventions must occur as this is dependent on local resources, but it is expected that the majority of patients will complete the study within 6 weeks of enrolment (table 1A,B,C).

**Table 1A:** Participant timeline in the study: the timeline for men randomised to TRUS biopsy

	Contact with patient				
	Visit 0*	Visit 1	Visit 2	Visit 3	Visit 4
Weeks:	−1	0	1	2	6
Teleconsult	X		Not required		
Consent		X			
Screening		X			
Randomisation		X			
EQ-5D-5L		X		X	X
Optional urine, blood and semen samples		X			
TRUS biopsy				X	
MRI					
MPMRI-targeted biopsy					
Immediate post-MRI questionnaire					
Immediate post biopsy questionnaire				X	
Follow-up for results of tests					X
Treatment decision†					X
30-day post biopsy questionnaire					X
30-day post-MRI questionnaire					
SAE	Complete as required at any time following registration				
Withdrawal form	Complete as required at any time following registration				

*This visit is optional depending on local site referral procedures. It is carried out over the phone 1 week prior to the first face-to-face visit.

†After treatment decision men revert to standard of care.

MPMRI, multi-parametric MRI; SAE, serious adverse event, TRUS, transrectal ultrasound-guided.

**Table 1B:** Participant timeline in the study: the timeline for men randomised to MPMRI who require an MPMRI-targeted biopsy

	Contact with patient				
	Visit 0*	Visit 1	Visit 2	Visit 3	Visit 4
Weeks:	−1	0	1	2	6
Teleconsult	X				
Consent		X			
Screening		X			
Randomisation		X			
EQ-5D-5L		X	X	X	X
Optional urine, blood and semen samples		X			
TRUS biopsy					
MRI			X		
MPMRI-targeted biopsy				X	
Immediate post-MRI questionnaire			X		
Immediate post biopsy questionnaire				X	
Follow-up for results of tests					X
Treatment decision†					X
30-day post biopsy questionnaire					X
30-day post-MRI questionnaire					
SAE	Complete as required at any time following registration				
Withdrawal form	Complete as required at any time following registration				

*This visit is optional depending on local site referral procedures. It is carried out over the phone 1 week prior to the first face-to-face visit.

†After treatment decision men revert to standard of care.

MPMRI, multi-parametric MRI; SAE, serious adverse event, TRUS, transrectal ultrasound-guided.

**Table 1C:** Participant timeline in the study: the timeline for men randomised to MPMRI who have no suspicious areas on MRI and do not require a biopsy

	Contact with patient				
	Visit 0*	Visit 1	Visit 2	Visit 3	Visit 4
Weeks:	−1	0	1	2	5
Teleconsult	X			Not required	
Consent		X			
Screening		X			
Randomisation		X			
EQ-5D-5L		X	X		X
Optional urine, blood and semen samples		X			
TRUS biopsy					
MRI			X		
MPMRI-targeted biopsy					
Immediate post-MRI questionnaire			X		
Immediate post biopsy questionnaire					
Follow-up for results of tests					X
Treatment decision†					X
30-day post biopsy questionnaire					X
30-day post-MRI questionnaire					X
SAE	Complete as required at any time following registration				
Withdrawal form	Complete as required at any time following registration				

*This visit is optional depending on local site referral procedures. It is carried out over the phone 1 week prior to the first face-to-face visit.

†After treatment decision men revert to standard of care.

MPMRI, multi-parametric MRI; SAE, serious adverse event, TRUS, transrectal ultrasound-guided.

#### Interventions—strategies to improve adherence to intervention protocols

After the treatment decision has been made, clinicians are permitted to order the test that the patient was not randomised to and participants are aware of this when enrolling in the study.

Participants are given reminders by the trial teams for completion of questionnaires at the correct time.

#### Interventions—concomitant care

Relevant anticoagulant/antiplatelet medication will be discontinued up to 10 days before biopsy and advice sought as to appropriate substitutes if indicated. Aspirin will be continued at the discretion of the physician doing the biopsy.

Participants may otherwise continue with any other concomitant care that they were receiving prior to enrolling on the study.

#### Withdrawal of individual participants

Participants can discontinue the participation of the study at any time for any reason. Data up to the time of withdrawal can be included in the study.

### Outcomes

#### Primary outcome

Proportion of men with clinically significant cancer detected. This will be evaluated on a per patient basis from needle biopsy. The primary definition will be a single core containing Gleason grade 3+4 disease or greater.

Time frame for assessment: when histology results are available, at an expected average of 30 days post intervention.

#### Secondary outcomes

Please see table 2 for a full list of secondary outcomes.

**Table 2:** Secondary outcomes in PRECISION

Outcome	Time frame for assessment
Proportion of men with clinically insignificant cancer (Gleason grade 3+3) detected	When histology results available, at an expected average of 30 days post intervention
Proportion of men in the MPMRI arm who avoid biopsy	When MRI results available, at an expected average of 30 days post-MRI
Proportion of men in whom the MPMRI score for suspicion of clinically significant cancer was 3, 4 or 5 but no clinically significant cancer was detected	When histology results available, at an expected average of 30 days post biopsy
Proportion of men who go on to definitive local treatment (eg, radical prostatectomy, radiotherapy, brachytherapy) or systemic treatment (eg, hormone therapy, chemotherapy)	After treatment decision, at an expected average of 30 days post biopsy
Cancer core length of the most involved biopsy core (maximum cancer core length, mm)	When histology results available, at an expected average of 30 days post intervention
Proportion of men with post biopsy adverse events	30 days post biopsy
Health-related quality of life	Baseline, 24 hours post intervention and 30 days post intervention
Proportion Gleason grade upgrading in men undergoing radical prostatectomy	An expected average of 90 days post biopsy
Cost per diagnosis of cancer	30 days post biopsy

MPMRI, multi-parametric MRI.

#### Sample size

Rates of clinically significant cancer detection from targeted-alone biopsy in a population with no prior biopsy have been shown to be 50%.^11^ Assuming 20% of men avoid biopsy in the MRI arm of PRECISION, this would correspond to a 50% detection rate in 80% of the participants in this arm =40% overall detection rate of clinically significant cancer in the MRI arm.

Rates of clinically significant cancer detection from one of the largest studies of TRUS biopsy in men without prior biopsy are shown to be 27%.^12^

For the non-inferiority hypothesis, using 90% power and 2.5% one-sided α, using an estimate for detection rate of clinically significant cancer for targeted biopsy of 40% and an estimate of detection rate for TRUS biopsy of 30% and using a margin of clinical unimportance of 5%, 211 men per arm will be required. The choice of 5% as the margin of non-inferiority represents a difference that would be considered clinically unimportant in the detection rates.

Thus the total men required in the study is 422.

Accounting for 10% withdrawal/loss to follow-up, 470 men will need to be recruited.

#### Recruitment

Enrolment will take place at the outpatient clinics of the participating medical centres. Prior to being approved to take part in the study, participating sites are required to confirm that they have enough eligible patients attending their centre to be able to recruit approximately three men per month. With at least 11 participating centres, it is estimated that the study will complete within 26 months of commencement. The start date of the study is February 2016 and the estimated completion date for recruitment is April 2018.

#### Randomisation and treatment allocation

Participants are randomised in a 1:1 ratio to either MPMRI or TRUS biopsy. Randomisation will be executed per participant, by web-based block randomisation after the informed consent procedure is completed. The University College London (UCL) Surgical and Interventional Trials Unit will facilitate the randomisation.

#### Sequence generation

Randomisation schedules are be prepared by a member of the UCL Surgical and Interventional Trials Unit using a bespoke SAS programme, with equal allocation between treatment arms using random permuted blocks of varying size, stratified by centre.

#### Allocation concealment

Only authorised staff members within the UCL Surgical and Interventional Trials Unit and the authorised staff member who uploads the sequence to the web-based randomisation service have access to the allocation sequence. None of these staff members are involved in clinical patient contact. The investigators responsible for patient recruitment who do have clinical patient contact do not have access to the sequence and are unaware of the block sizes. The allocation for the next patient is given on a patient by patient basis by an authorised staff member or electronically by the web-based system.

#### Blinding

In this trial, blinding of the participant or the clinician is not feasible. While the study is progressing, for any clinical outcomes that come to the attention of the PRECISION Operations Group (eg, serious adverse events (SAEs)), the main study statistician will be blinded as to which group these outcomes are from. Where feasible, when analysing the outcome data from the study, the study statistician will be blinded to the groups until the end of the analysis.

#### Data management

To ensure high quality trial conduct, data management will be carried out according to the principles of the International Council of Harmonisation Good Clinical Practice. Data will be entered onto a web-based MARVIN e-case report form (eCRF) system which increases data quality. Quality control is carried out routinely; data types, entries and permitted ranges for answers to every question on the eCRFs are restricted on this web-based system. Automatic validation checks and automatic queries are raised by the system immediately to individual sites in the case of any queries. Authorised individuals from the PRECISION Operations Group may also check the data for quality and may pose manual queries to the site to address. A proportion of participating sites’ MRI and histopathology data may be verified centrally.

The data will be stored by the PRECISION Operations Group for 20 years after the final publication from the trial. Further details on data storage, curation and destruction are available in a separate document on request to the UCL Surgical and Interventional Trials Unit.

#### Data collection methods

An eCRF user guide has been produced to aid sites in completing all eCRFs. Sites are required to confirm in writing that they have practised entering imaginary patient data for all eCRFs into a demo version of the MARVIN eCRF system prior to being granted approval to use the live MARVIN eCRF system. Only authorised users of the system on the local site delegation log have access to the MARVIN system. Details on how to collect data in the study are given in a mandatory site initiation visit. The content of the presentation must be viewed by all local site staff.

Baseline patient and follow-up data will be recorded by authorised individuals from clinical encounters during the study. Self-reported, previously validated EQ-5D-5L questionnaires will be used to assess health-related quality of life.^13^ Post biopsy complications will be assessed by self-reported questionnaires based on previously validated questionnaires.^14^

#### Participant retention

Once a participant is enrolled or randomised, the local site will make every reasonable effort to follow the patient for the entire study period. It is estimated that loss to follow-up/withdrawal after randomisation will be no more than 10%. Study site staff are responsible for developing and implementing local standard operating procedures to achieve this.

#### Statistical methods

The formal statistical analysis plan will be finalised before database lock and before any statistical analysis.

A consort diagram will be presented. All continuous variables will be described using the mean, SD, median, minimum and maximum. Categorical variables will be described using frequencies and proportions.

The statistical assumptions underpinning each method will be checked. The use of transformations or non-parametric methods will be considered to satisfy statistical assumptions.

#### Primary outcome analysis

The primary analysis will be based on an intention to treat sample as well as a per protocol sample.

Our primary end point is the difference in proportion of men with clinically significant cancer detected between the two groups.

Absolute differences in proportion of clinically significant cancer detected between arms will be calculated with 95% CIs.

If the lower bound of the 97.5% CI for the difference in detection rates of MPMRI+/- targeted biopsy compared with TRUS biopsy is greater than −5% then MPMRI+/-- targeted biopsy will be deemed non-inferior.

In the event that the lower bound is greater than zero, superiority can be claimed.

Baseline characteristics will be compared between the two groups. Any imbalance found between the groups will be accounted for in a multivariable logistic regression analysis. This model will include clinically significant cancer as the dependant variable, group as the independent variable and any characteristics with baseline imbalance as a covariate. Potential confounders identified a priori will also be considered in the model described here. Here, the effect of centre will also be explored using a mixed logistic regression model with clinically significant cancer as the dependant variable, group as the independent variable and centre as a random effect.

The effect of missing data will be explored and dealt with in an appropriate manner.

#### Secondary outcome analyses

The effect sizes will be presented with 95% CIs.

For binary outcomes, absolute differences in proportion between arms will be calculated with 95% CIs. Any imbalance found between the groups will be accounted for in a multivariable logistic regression analysis.

For continuous outcomes, mean differences in outcomes between arms will be calculated with 95% CIs. Any imbalance found between the groups will be accounted for in a multivariable linear regression analysis. Where the outcomes are not normally distributed, a suitable transformation or non-parametric test will be considered.

#### Descriptive analysis

Where a patient after receiving the per protocol treatment receives another test which leads to further knowledge about the presence or absence of cancer, this will be described. The number of false positives and false negatives will be recorded.

#### Monitoring

The Global Trial Steering Committee (GTSC) consists of a team independent of the PRECISION Operations Group and independent of the principal investigators (PIs). It comprises chairs, advisory board members and chief investigators. Observers from the UCL Surgical and Interventional Trials Unit are present during meetings. The GTSC’s role is to:

1. To monitor and supervise the progress of the study towards its overall objectives at regular intervals
2. To consider the recommendations of the Data Monitoring Committee (DMC) and make recommendations on future study conduct

The DMC consists of an independent chair, clinical representative, patient representative and statistician. The DMC’s role is to safeguard the interests of trial participants and monitor the overall conduct of the clinical trial. The DMC is independent of, but reports to, the GTSC. They will meet at regular intervals throughout the study period as determined by the chair, but as a minimum at least once every 12 months.

The DMC Charter can be obtained by request to the Surgical and Interventional Trials Unit.

The sponsor may also arrange an independent trial monitor to audit the study data.

#### Harms

AEs will be defined as ’any untoward medical occurrence in a clinical trial subject undergoing any intervention in the trial, which does not necessarily have a causal relationship with this treatment’.

SAEs will be defined as ’any untoward medical occurrence as a result of any intervention in the trial that:

- results in death,
- is life-threatening
- requires hospitalisation or prolongation of existing inpatients' hospitalisation, results in persistent or significant disability or incapacity’

AEs and SAEs will be recorded until 30 days post biopsy. In the event that the patient does not undergo biopsy, AEs and SAEs should be recorded until 30 days post-MRI.

AEs will be recorded by a member of the research team or clinical team on an AE report form eCRF. All SAEs must be recorded on an SAE report form eCRF within 24 hours of knowledge of the SAE. Both AEs and SAEs should be recorded in the medical notes. Completed AE and SAE report forms should be sent to the UCL Surgical and Interventional Trials Unit, who will keep a log of AEs and SAEs. AE and SAE logs will be reviewed by the DMC.

#### Ethics and dissemination

The protocol and each participating site will have Research Ethics Committee (REC) approval before participants are entered. The UK National REC (NRES Committee East Midlands, Leicester) gave favourable approval for study on 3 June 2015 (Ref:15/EM/0188).

Amendments to the protocol will be made after ethical approval by the research ethics committee in each respective centre. Centres will be notified immediately after any protocol amendments.

#### Consent

The clinical teams managing patients with suspected prostate cancer who are referred to their centre will identify potential trial participants. Patient information sheets will be provided to patients. Members of staff who are trained to take written informed consent, as indicated by the PI on the delegation log for that site, will take written informed consent in a face-to-face visit. A model consent form is shown in online supplementary appendix 1 and a model patient information sheet is shown in online supplementary appendix 2.

10.1136/bmjopen-2017-017863.supp1 Supplementary file 1

Additional ethics-approved consent will be sought from patients for collection of biological samples (blood, urine, semen), pending additional funding being secured for collection and processing of samples. If funding is secured, these samples will be stored in the UCL/Royal Free Biobank for use in future biomarker studies (supplementary appendix 3).

#### Confidentiality

The data of the participants will be recorded into the eCRF system and analysed without any personal identifiers, by using coded information. The source documents and identification lists will be archived in a secured facility per centre. Permission for accessing data will be documented per investigator.

#### Dissemination

Results of this study will be disseminated through national and international conferences and papers. Authorship criteria will be based on recommendations of the International Committee of Medical Journal Editors. The participants and relevant patient support groups will be informed about the results of the study.

#### Access to data

Only authorised individuals within the PRECISION Operations Group have access to the final data set. Individual PIs have access to their own data but not that of other sites.

#### Declaration of interests

ME has stock holding in NUADA Medical (as a founder member). NUADA Medical is a healthcare company specialising in urology and gynaecology. They provide a range of imaging including MRI of the prostate. The PRECISION Operations Group reports no other interests to declare. Declaration of interests will be ascertained from each PI participating in the study and reported in full on dissemination of study results.

### WHO trial registration data set

For the WHO trial registration data set please see table 3.

**Table 3:** WHO trial registration data set

Data category	Information
Primary registry and trial identifying number	ClinicalTrials.gov: NCT02380027
Date of registration in the primary registry	23 February 2015
Secondary identifying numbers	ISRCTN: 18440098
Source(s) of monetary or material support	National Institute for Health and Research UK (DRF-2014-07-146) European Association of Urology Research Foundation
Primary sponsor	University College London
Secondary sponsor(s)	N/A
Contact for public queries	Mr Veeru Kasivisvanathan veeru.kasi@ucl.ac.uk Urology Research Group, Room 4.23, Fourth floor, 132 Hampstead Road, London, NW1 2PT Tel:+44 (0)207 679 9092, Fax:+44 (0)207 679 9511
Contact for scientific queries	Mr Veeru Kasivisvanathan veeru.kasi@ucl.ac.uk Urology Research Group, Room 4.23, Fourth floor, 132 Hampstead Road, London, NW1 2PT Tel:+44 (0)207 679 9092, Fax:+44 (0)207 679 9511
Public title/short title	Prostate evaluation for clinically important disease: sampling using image guidance or not?
Acronym	PRECISION
Scientific title	A randomised control trial of MRI-targeted biopsy compared with standard TRUS biopsy for the diagnosis of prostate cancer in men without prior biopsy
Countries of recruitment	Argentina Belgium Canada Finland France Italy Germany Netherlands Switzerland UK USA
Health condition(s) or problem(s) studied	Prostate neoplasm
Intervention(s)	Device: MRI. This will be a multi-parametric MRI of the prostate. Procedure: MRI-targeted biopsy. This will be a biopsy targeted to suspicious areas on the MRI. Procedure: TRUS biopsy. This will be a standard 12-core transrectal prostate biopsy.
Intervention description	1. Experimental: MRI-arm Men in this arm will undergo multi-parametric MRI. In the presence of a suspicious area, a man will undergo MRI-targeted biopsy with cores targeted to the suspicious lesion. In the absence of a suspicious area, no biopsy will be taken. Interventions: Device: MRI Procedure: MRI-targeted biopsy 2. Active comparator: TRUS biopsy arm Men in this arm undergo standard 12-core TRUS prostate biopsy. Intervention: Procedure: TRUS biopsy
Key inclusion and exclusion criteria	Inclusion criteria: Men at least 18 years of age referred with clinical suspicion of prostate cancer who have been advised to have a prostate biopsy Serum PSA ≤20 ng/mL within the previous 3 months Suspected stage ≤T2 on rectal examination (organ-confined prostate cancer) within the previous 3 months Fit to undergo all procedures listed in the protocol Able to provide written informed consent
	Exclusion criteria: Prior prostate biopsy Prior treatment for prostate cancer Contraindication to MRI (eg, claustrophobia, pacemaker, estimated glomerular filtration rate ≤50 mls/min) Contraindication to prostate biopsy Men in whom artefact would reduce the quality of the MRI Previous hip replacement surgery, metallic hip replacement or extensive pelvic orthopaedic metal work Unfit to undergo any procedures listed in the protocol
Study type	Interventional Allocation: randomised Intervention model: parallel assignment Masking: statistician Primary purpose: diagnostic Sequence generation: bespoke SAS programme Block randomisation Achievement of allocation concealment: only authorised staff members have access to the allocation sequence. No investigators have access to the sequence. The allocation for the next patient is given by an authorised staff member or electronically after the investigator confirms that the eligibility criteria for inclusion of that patient have been met.
Date of first enrolment	10 February 2016
Target sample size	470
Recruitment status	Recruiting
Primary outcome(s)	Proportion of men with clinically significant detected
Key secondary outcomes	Proportion of men with clinically insignificant cancer detected (time frame: when histology results are available, at an expected average of 30 days post biopsy) Proportion of men in the MRI arm who avoid biopsy (time frame: when MRI results are available, at an expected average of 30 days post-MRI) Proportion of men with an MRI score 3, 4 or 5 who have no clinically significant cancer detected (time frame: when histology results are available, at an expected average of 30 days post biopsy) Proportion of men who go on to definitive treatment for prostate cancer (time frame: after treatment decision, at an expected average of 30 days post biopsy) Definitive treatment can be localised (eg, radical prostatectomy, radiotherapy, brachytherapy) or systemic (hormone therapy, chemotherapy) Cancer core length in mm of the most involved biopsy core (maximum cancer core length) (time frame: when histology results available, at an expected average of 30 days post biopsy) Proportion of men with post biopsy adverse events (Time frame: 30 days post biopsy) EQ-5D-5L Quality of Life Scores (time frame: baseline, 24 hours post intervention and 30 days post intervention) Proportion of men undergoing radical prostatectomy who have Gleason grade upgrading (time frame: an expected average of 90 days post biopsy) Cost per diagnosis of cancer (time frame: 30 days post biopsy)

PSA, prostate-specific antigen; TRUS, transrectal ultrasound-guided.

### Current protocol version

The current protocol is V.1.2, issued 26 August 2015. The current protocol amendment number is 02. For full amendment history please see table 4.

**Table 4:** Revision chronology for amendments to protocol

Protocol version and date	Reasons for amendments
V.1.0, issued 11 April 2015	Original protocol
V.1.1, issued 24 May 2015	Main reasons for amendment: minor changes in the patient information sheet to ensure compliance with research ethics committee regulations. Main changes: Version number and date added to all pages Patient advice and liaison service contact details specified Sentence changed from ‘different treatment’ to ‘a different diagnostic test’ The name of the approving research ethics committee added ‘NRES Committee East Midlands - Leicester’
V.1.2, issued 26 August 2015	Main reasons for amendment: minor changes to make existing trial documents clearer. Main changes: International Standard Registered Clinical/soCial sTudy Number (ICRCTN) number and UK Clinical Research Network(CRN) identifiers added. Wording in Section 17.3 Assessment of Safety. Wording clarified to ensure it is clear which expected adverse events do not need to be reported. Updates of sites and PIs participating in the trial. Clarification in TRUS biopsy conduct section 11.2. TRUS biopsy as described in the protocol allows 10–12 cores to be taken. Although this was clear in the protocol, this is clarified throughout the text now as the procedure is often quoted as 12-core biopsy, which may otherwise be confusing to the PI. Appendix 7—EQ-5D—update of copyright date from 1990 to 2009. No change in content. In the ‘What will happen to me if I take part’ section, clarification that the timelines are suggested timelines and that timelines for trial procedures depend on clinical workload at the hospital.

PI, principal investigator; TRUS, transrectal ultrasound-guided.

### Funding

This work was funded by a doctoral research fellowship for VK from the National Institute for Health Research (DRF-2014-07-146). UK sites are funded by inclusion of the study in the NIHR portfolio (UKCRN ID 18902 PRECISION). Non-UK sites are funded by the European Association of Urology Research Foundation (EAURF2015001). The design, management, analysis and reporting of the study are entirely independent of the funders of the study.

### Roles and responsibilities

For roles and responsibilities of the trial sponsor and committees involved in the study, see table 5.

**Table 5:** Roles and responsibilities in the PRECISION Trial

Role	Details and responsibilities
Trial sponsor	University College London (UCL) Sponsor’s reference: UCL REDA Number 15/0299 Contact name: Susan Tebbs Address: Comprehensive Clinical Trials Unit (CCTU) UCL, Gower Street, London, WC1E 6BT Telephone: 020 3447 5557 Email: Rand.D@uclh.nhs.uk The trial sponsor did not provide any funding for the study. University College London has the role of research governance sponsor of PRECISION. UCL adopted the study as sponsor after the UCL CCTU carried out a trial adoption process which involved the UCL CCTU reviewing the protocol to ensure it conformed to high standards of trial conduct and met the governance requirements of UCL. The UCL CCTU is responsible for oversight of the trial. The sponsor plays no role in data collection, management, analysis and interpretation of data, writing of the report or the decision to submit the report for publication.
PRECISION Operations Group	The PRECISION Operations Group consists of the chief investigators, the study coordinator, the UCL Surgical and Interventional Trials Unit and the eCRF database managers. This group is responsible for: Study planning Preparation of protocol and revisions Assistance with international review board/independent ethics committee applications Preparation of investigators brochure and CRFs Organisation of steering committee meetings Provide annual progress reports to the ethics committee Reporting serious adverse events to the sponsor and ethics committee when necessary Responsible for trial master file Budget administration and contractual issues with individual centres Advice for PIs Site initiation visits Randomisation Data verification and management Central monitoring and resolving data queries with clinicians and nurses at the trial sites Maintenance of the trial Information Technology (IT) system Publication of study reports
PI	At each participating site, the PI is responsible for the conduct of the clinical trial to ensure the safety of participants and the reliability and robustness of the data generated. They will be responsible for identification, recruitment, data collection and completion of CRFs, along with follow-up of study patients and adherence to study protocol and investigators brochure. The PI as leader of the research team may delegate his/her duties to members of his/her team.
GTSC	Consists of a team independent of the PRECISION Operations Group and independent of the PIs. It comprises independent chairs, advisory board members and chief investigators. Observers from the Clinical Trials Group are present during meetings. The GTSC’s role is to: 1. To monitor and supervise the progress of the study towards its overall objectives at regular intervals 2. To consider the recommendations of the DMC and make recommendations on future study conduct
DMC	Consists of an independent chair, clinical representative, patient representative and statistician. The DMC’s role is to safeguard the interests of trial participants and monitor the overall conduct of the clinical trial. The DMC is independent of, but reports to, the GTSC.

CRF, case report form; DMC, Data Monitoring Committee; GTSC, Global Trial Steering Committee; PI, principal investigator.

## Supplementary Material

Reviewer comments
