# Supplementary material for: A multicentre randomised controlled trial assessing whether MRI-targeted biopsy is non-inferior to standard transrectal ultrasound guided biopsy for the diagnosis of clinically significant prostate cancer in men without prior biopsy: a study protocol
Source: BMJ Open. 2017 Oct 12;7(10):e017863. doi: 10.1136/bmjopen-2017-017863 (PMC5706484; doi:10.1136/bmjopen-2017-017863)
Supplement: Supplementary file 1 [file bmjopen-2017-017863supp001.pdf]

Appendix 1: Model consent form

(To be presented on local headed paper)

REC Number: 15/EM/0188   Centre Number

Subject Identification Number:

CONSENT FORM

Title of Project PRostate Evaluation for Clinically Important disease: Sampling using Image-guidance Or Not. (PRECISION)

Name of Researcher:

Please initial box

1. I confirm that I have read and understand the information sheet dated................... (version...........) for the above study. I have had the opportunity to consider the information, ask questions and have had these answered satisfactorily.

2. I understand that my participation is voluntary and that I am free to withdraw at any time without giving any reason, without my medical care or legal rights being affected.

3. I understand that relevant sections of my medical notes and data collected during the study, may be looked at by individuals from the sponsor of the trial (University College London) and responsible persons authorised by the sponsor, from regulatory authorities or from the NHS Trust, where it is relevant to my taking part in this research. I give permission for these individuals to have access to my records.

4. I agree to my GP being informed of my participation in the study.

5. I agree to take part in the above study.

All boxes above must be initialed for consent to be valid

The following requests are optional, if you do not give permission, you can still participate in the study

6. I agree to giving urine and/or blood and/or semen samples and/or prostate pathology specimens which will be stored and made available for future ethically approved research ☐

7. After I complete my involvement in the study, I give permission for the research team to check my records through the Office of National Statistics and NHS databases and I give permission to be contacted for further information ☐

8. I give permission for my full postal, email address and mobile phone number to be recorded and stored. This will be used for research purposes only and will remain confidential. This information may be used for sending out quality of life questionnaires, if required. ☐

Name of Participant	Date	Signature
Name of Person taking consent	Date	Signature
Name of Chief Investigator *(if different to the person taking consent)*	Date	Signature

When completed: 1 for participant; 1 (original) for researcher site file; 1 to be kept in medical notes.

Appendix 2: Model patient information sheet

PLACE HOSPITAL LETTER HEAD ON FIRST PAGE ONLY.

Affix patient sticker / details here

Version 1.2, 26th August 2015

This is the Patient Information Sheet for a Health Research Study called PRECISION

Study Short Title:
PRostate Evaluation for Clinically Important disease: Sampling using Image-guidance Or Not

Study acronym:
PRECISION

Chief Investigators: Mrs Caroline Moore, Professor Mark Emberton
Study coordinator: Mr Veeru Kasivisvanathan

UCL Reference number: 15/0299

REC Reference number: 15/EM/0188

We would like to invite you to take part in our research study. Before you decide we would like you to understand why you are being invited, why the research is being done and what it would involve for you. One of our team will go through the information sheet with you and answer any questions you have. Talk to others about the study if you wish.

Part 1 tells you the purpose of this study and what will happen to you if you take part.
Part 2 gives you more detailed information about the conduct of the study.

Ask us if there is anything that is not clear. You will have at least 24 hours to decide whether or not to take part but take as much time as you need to consider the study.

Part 1

1. Why have I been invited?

You are being invited because you may require further investigation of your prostate with a prostate biopsy. You have not been diagnosed with cancer but a biopsy may be required to establish whether you do or do not have cancer. The clinical Urology team that you have been referred to has informed us that you may be eligible for this study.

2. What is the purpose of the study?

The standard way of diagnosing prostate cancer is to carry out a trans-rectal ultrasound guided (TRUS) biopsy. This involves inserting an ultrasound probe into the back passage after which 10-12 pieces of tissue are taken from the prostate from areas in the prostate most likely to contain cancer. Another way of doing a biopsy is to perform an MRI scan of the prostate on an earlier day and use that information to help take the biopsies. If there is a suspicious area in the prostate on the MRI, a few biopsies can be directed at where the suspicious area is thought to be, also using a probe in the back passage. Up to 12 pieces of tissue can be taken. If there is no suspicious area on the MRI, which occurs in about 30% of men, then no biopsy will be taken at all.

We currently do not know for certain whether using MRI directed biopsies will allow us to detect the same, more or less prostate cancer than if we do not use MRI. Current evidence supports the idea that using MRI directed biopsies may detect a similar amount of cancer to when it is not used but one advantage is it may allow a man to avoid a biopsy.

The main purpose of this study is to assess whether MRI-targeted biopsy can detect a similar amount of cancer as 10-12-core TRUS biopsy.

3. Do I have to take part?

It is up to you to decide to join the study. We will describe the study and go through this information sheet. If you agree to take part, we will then ask you to sign a consent form. You are free to withdraw at any time without giving a reason. This will not affect the standard of care you receive.

4. What are the benefits to me of taking part in this study?

You will benefit by having the diagnostic test that will help us establish whether or not you have prostate cancer. The research team will ensure your tests are carried out as quickly as possible and will be a point of contact for you should you have any concerns or questions.

If you are required to have an MRI (50% of participants) then there is a chance (up to 30%) that you could avoid a biopsy and its risks altogether if the MRI is normal.

The information we get from this study will help improve the diagnosis of prostate cancer for men in the future.

5. What type of study is this?

This is a randomised study – Sometimes we don't know which way of investigating patients is best. To find out, we need to compare different tests. We put people into groups and give each group a different diagnostic test. The results are compared to see if one is better. To try to make sure the groups are the same to start with, each patient is put into a group by chance (randomly).

You will be required to attend a screening visit with a member of the research team who will spend around 40 minutes explaining what is involved in the study and making sure you are eligible for the study. You will be asked to fill out a short questionnaire.

You will then be randomly allocated to one of two groups. One group will receive a TRUS-biopsy and the other group will receive an MRI before the doctors decide on whether to perform a biopsy or not. If a biopsy is required after the MRI then it will be carried out taking into account information from the MRI.

6. What will happen to me if I take part?

In this study you will asked to visit the hospital 3-4 times in total depending on which group you are allocated to. This is a similar number of times to if you were not taking part in the study.

If you are assigned to the TRUS-biopsy group you will then undergo the TRUS-biopsy approximately at 2 weeks following the screening visit. The procedure itself takes about 40 minutes and is usually carried out under local anaesthetic. Typically 12 cores are taken. We wait for the results and discuss treatment options with you in clinic at around 2-3 weeks after the biopsies. You will be required to fill out questionnaires after the biopsies and at 30 days after the biopsies. The questionnaires should take about 10 minutes to fill out and you can post them back to us. You may be reminded to fill the questionnaires out by a phone call from the research team.

If you are assigned to the MRI group, you will have an MRI at about 2-3 weeks of the screening visit. The MRI takes about 40 minutes. You will need to fill in a questionnaire after this. If you have an MRI with a high enough suspicion (MRI Score 3, 4 or 5) you will be booked for a biopsy following the MRI.

The biopsy procedure itself takes about 40 minutes and is usually carried out under local anaesthetic. A maximum of 12 cores will be taken but this may be fewer. It will be carried out using the MRI scan to influence where the biopsies are taken from. Software may be used to transfer additional information from the original MRI onto the screen when the biopsies are taken.

We then wait for the results and discuss treatment options with you for 30 minutes in clinic approximately at 2-3 weeks after the biopsies. You will be required to fill out a questionnaire after the biopsies and at 30 days after the biopsies. If you do not need a biopsy then we will explain this to you approximately 2 weeks following your MRI and you will need to complete a questionnaire 30 days after the MRI. You may be reminded to fill the questionnaires out by a phone call from the research team. You can post these questionnaires back to us.

Please note that the above time frames are suggested time frames and depending on clinical workload within the hospital, the time frame may be longer. This would be no different than if you were not part of the study.

As an additional option you will be asked at the consent and screening visit if you are happy to provide urine, semen and blood samples which will be stored for future research studies looking into identifying markers within these substances that can help us diagnose prostate cancer better. You will also be asked if the prostate tissue from your biopsies, after they have been analysed for your care, can be stored for these future research studies. This is optional and not a necessity to take part in the study.

Being involved in the study does not limit subsequent tests or treatment you may receive. If you do undergo further tests or treatment after the study is complete we may check the results of these on your records. After completing the study, we also ask your permission to check your health through national databases. All information which is collected about you during the course of the research will be kept strictly confidential, and any information about you which leaves the hospital will have your name and address removed so that you cannot be recognised. Please see Part 2 for further information on this.

Study Schema:

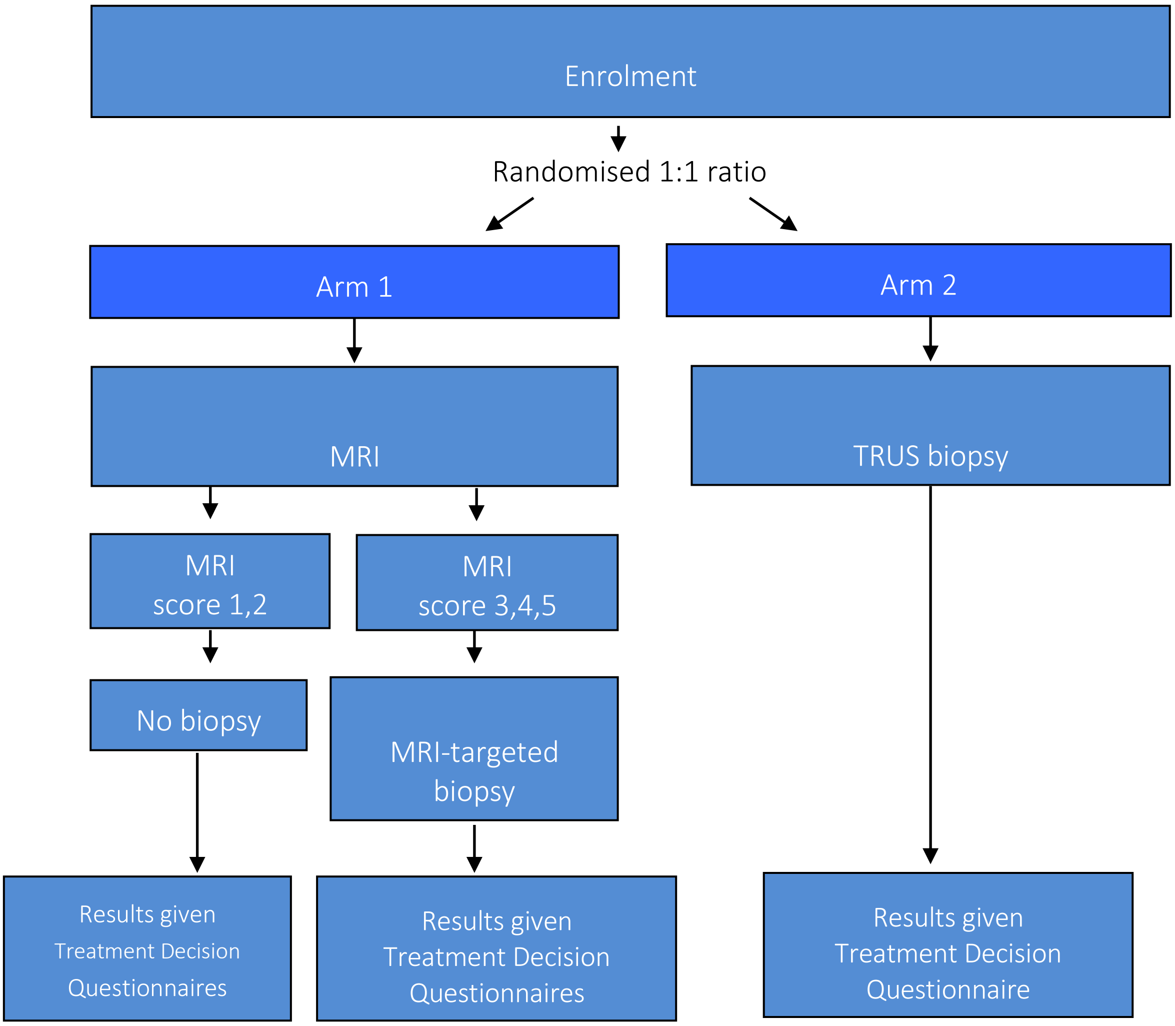

7. What will I have to do?

You should attend your screening visit and if eligible for the study, await contact from the hospital for further dates of investigations. Unless otherwise advised by a doctor you should carry on with you normal activities and medication. Sometimes before a biopsy your doctor will prescribe you antibiotics and may ask you to stop blood-thinning medications.

You should undergo the necessary tests and biopsy procedures that you are advised to have by your doctor.

You will be required to fill out questionnaires immediately after the biopsies and during follow up. You should attend your follow up clinic appointment where we discuss your results. Treatment options will be discussed with you at the results clinic. In total you will be required to attend the hospital 3-4 times.

8. What are the alternatives for diagnosis?

The diagnosis of prostate disease usually requires a prostate biopsy. All prostate biopsies involve the use of a biopsy device to take samples from the prostate gland. MRI is an additional test to help diagnose prostate cancer. If an MRI shows a suspicious area, biopsies will still be required to diagnose prostate cancer. If an MRI does not show a suspicious area, in some cases men may be able to avoid a biopsy.

9. What are the possible disadvantages and risks of taking part?

Being involved in the study puts the participants at no additional risk than if they were not involved in the study but underwent the normal procedures for men referred for further investigation of prostate disease.

Risks of prostate biopsy include:

- Temporary discomfort in the back passage (most men)
- Blood in the urine – up to 2 weeks (most men)
- Blood in the semen – up to 3 months (most men)
- Blood in the back passage – up to 1 week (most men)
- Infection in the blood stream – 1-4 out of 100 men
- Urinary tract infection – 4 out of 100 men
- Urinary retention – 1 out of 100 men
- Adverse reaction to antibiotics – less than 1 in 100 men

Risks of MRI include:

- Discomfort from cannulation
- Allergic reaction:
    - Mild reaction e.g. rash, itching – less than 1 in 250 men
    - Moderate reaction e.g. nausea, omitting – less than 1 in 2000 men
    - Severe reaction e.g. breathing problems – less than 1 in 10000 men

It is not known which of the two biopsy techniques (TRUS-biopsy or MRI-targeted biopsy) is more effective, though current evidence suggests that they are similar. Both biopsy techniques are used routinely in everyday clinical practice.

Before participating you should consider if this will affect any insurance you have and seek advice if necessary.

10. What should you do if you experience any problems during the study?

Though the risk is very low, if you do experience any possible signs of infection after biopsies (fevers and feeling generally unwell) then you should urgently go to your nearest accident and emergency department which is open 24 hours a day. If you are not able to pass urine you should urgently go to your nearest accident and emergency. If you are unsure about what to do or have any questions please call 0207 679 9092 between 9am and 5pm and a member of our research team may be able to offer you advice.

If you experience any side effects after biopsy as listed in the 30-day questionnaire please record these in the relevant section in the questionnaire. If you experience any other untoward complication or need to see a doctor we would like to know about this so please let us know on the above number as soon as possible after the complication. For any emergencies at any time or if you are unable to contact a member of the research team, please attend your local accident and emergency for an assessment.

11. What happens when the research study stops?

Once the results of the biopsy are available you will be called to clinic to discuss them. Once a treatment decision is made, most men in the study will complete the study and your normal clinical team will continue to look after your care. Being part of the study does not prevent you from undergoing any further diagnostic test or treatment that your clinician would normally recommend.

12. What if there is a problem?

Any complaint about the way you have been dealt with during the clinical study or any possible harm you might suffer will be addressed. The detailed information concerning this is given in Part 2 of this information sheet. If you have any concerns or complaints you should contact a member of the research team in the first instance.

13. Will my taking part in the study be kept confidential?

Yes. We will follow ethical and legal practice and all information about you will be handled in confidence. The details are included in Part 2.

14. Will any costs I incur in travelling to study visits be reimbursed to me?

Reasonable transport costs that you incur to get to additional study visits that are above what you would normally need if you were not part of the study may be reimbursed. Please contact your local study nurse or doctor or the Study Coordinator (details below) for further information on claiming.

15. Contact Details

If you have any further questions or need any further information please do no hesitate to contact the research team:

Study Co-ordinator:
Mr Veeru Kasivisvanathan MBBS BSc MRCS MSc
Division of Surgery and Interventional Science, University College London

4th Floor, 132 Hampstead Road, London. NW1 2PS
T: 0207 679 9092 F: 0207 679 9511 E: veeru.kasi@ucl.ac.uk

Chief Investigators:
Mrs Caroline Moore MD FRCS
Division of Surgery and Interventional Science, University College London
4th Floor, 132 Hampstead Road, London. NW1 2PS
T: 0207 679 9092 F: 0207 679 9511 E: caroline.moore@ucl.ac.uk

Professor Mark Emberton MD FRCS
Division of Surgery and Interventional Science, University College London
4th Floor, 132 Hampstead Road, London. NW1 2PS
T: 0207 679 9092 F: 0207 679 9511 E: mark.emberton@ucl.ac.uk

This completes Part 1 of the Information Sheet.

If the information in Part 1 has interested you and you are considering participation, please read the additional information in Part 2 before making any decision.

Part 2

16. What if relevant new information becomes available?

Sometimes we get new information about the procedures being studied. If this happens, we will tell you about it and discuss whether you want to or should continue in the study. If you decide not to carry on, we will make arrangements for your care to continue. If you decide to continue in the study we will ask you to sign an updated consent form.

17. What will happen if I don't want to carry on with the study?

You can withdraw from the study at any point and it will not affect the care that you are given. We will use information collected about you up until your withdrawal. Kindly keep in contact with us to let us know your progress.

18. What if there is a problem?

If you have a concern about any aspect of this study, you should ask to speak to the research team who will do their best to answer your questions (0207 679 9092). You can also contact the Study Coordinator or Chief Investigators on the number or address given earlier in this document. If you wish to complain by other means or have any concerns about any aspect of the way you have been approached or treated by members of staff or about any side effects (adverse events) you may have experienced due to your participation in the clinical study, the normal National Health Service complaints mechanisms are available to you. You can contact the hospital Patient Advice and Liaison Service (PALS) at University College Hospital London on 020 3447 3042. If you have a different local hospital, their PALS number can be found on the NHS Choices Website at http://www.nhs.uk/chq/pages/1082.aspx?CategoryID=68. Further details can also be obtained from the Department of Health website: http://www.dh.gov.uk.

Every care will be taken in the course of this clinical study. However in the unlikely event that you are injured by taking part, compensation may be available.

If you suspect that the injury is the result of the Sponsor's (University College London) or the hospital's negligence then you may be able to claim compensation. After discussing with your study doctor, please make the claim in writing to Mrs Caroline Moore who is the Chief Investigator for the clinical study and is based at University College London. The Chief Investigator will then pass the claim to the Sponsor's Insurers, via the Sponsor's office. You may have to bear the costs of the legal action initially, and you should consult a lawyer about this.

Participants may also be able to claim compensation for injury caused by participation in this clinical study without the need to prove negligence on the part of University College London or another party. You should discuss this possibility with your study doctor in the same way as above.

19. Will my taking part in this study be kept confidential?

If you consent to take part in this study, the records obtained while you are in this study as well as related health records will remain strictly confidential at all times. The information will be held securely on paper and electronically at

your treating hospital under the provisions of the 1998 Data Protection Act. The information will be made available to persons in the clinical and research teams treating you. Your name and personal details will not be passed to anyone else outside the clinical team, research team or the Sponsor, who is not involved in the study. In some cases the research team may verify results of tests at University College London Hospital carried out at your local hospital (for example MRI results or prostate biopsy results).

Any data stored by the research team outside of your treating hospital will be kept at a secure location and will not contain information that can directly identify you. You will be allocated a study number, which will be used as a code to identify you on all study forms and data. The information will be linked to you so that if we did need to identify you for your safety or to clarify some information we would be able to by using a unique key, which will be known to the research team.

Your records will be available to people authorised to work on the study but may also need to be made available to people authorised by the Sponsor, which is the organisation responsible for ensuring that the study is carried out correctly. By signing the consent form you agree to this access for the current study and any further research that may be conducted in relation to it, even if you withdraw from the current study. All will have a duty of confidentiality to you as a research participant.

If you withdraw consent from further study treatment, your data and samples will remain on file and will be included in the final study analysis.

In line with the regulations, at the end of the study your data will be securely archived for a minimum of 5 years. Arrangements for confidential destruction will then be made.

Anonymised data collected during the study may be transferred for the purpose of processing or analysis to associated researchers within/outside the European Economic Area. Some countries outside Europe may not have laws which protect your privacy to the same extent as the Data Protection Act in the UK or European Law. The Sponsor of the study will take all reasonable steps to protect your privacy.

In the future we may publish our findings from the study in scientific journals but you will not be identifiable in any publications.

20. Will my GP be informed of my involvement?

Because this study is not being carried out by your GP we would like to inform him or her of your participation. If you agree to take part and agree to us contacting your GP, we will give him or her details of the study and inform them that you have chosen to participate in it. You will not be able to participate in this study if you do not give us this permission to inform your GP.

21. What will happen to any samples I give?

The majority of samples taken for this study are routinely carried out as part of the investigation of prostate disease. Samples include blood, urine and prostate tissue. The samples will be analysed and stored at the hospital site that they were carried out at as would normally be done in routine care. In some cases, a member of the research team at University College London Hospital may verify the prostate biopsy results. This is done routinely in clinical practice for some patients where results need to be confirmed by another doctor.

There is the additional option of giving urine, blood and semen samples and prostate tissue samples from biopsies specifically to be stored for future research purposes that are approved by a research ethics committee. These samples will be stored securely at a purpose-built facility. They will not be identifiable to you and you will not be informed if they are used in the future.

22. What will happen to the results of the research study?

The results of the study will be available after it finishes and will usually be published in a medical journal or be presented at a scientific conference. The data will be anonymous and it will not be possible to identify you in any report or publication.

Should you wish to see the results, or the publication, please ask your study doctor or visit www.ctgparticipant.org.

23. Who is organising and funding the research?

The study is funded by the National Institute for Health and Research (NIHR) UK. The study coordinator Mr Veeru Kasivisvanathan, is funded by a Doctoral Research Fellowship by the NIHR. The work may form part of his Doctoral Thesis.

24. Who has reviewed the study?

All research in the NHS is looked at by an independent group of people, called a Research Ethics Committee, to protect your interests. This study has been reviewed and given favourable opinion by National Research Ethics Service Committee East Midlands - Leicester. Patients and members of the public have also reviewed the study documents to ensure they are appropriate and well written.

25. Further information

You are encouraged to ask any questions you wish, before, during or after your investigations. If you have any questions about the study, please speak to your study nurse or doctor on the numbers specified below, who will be able to provide you with up to date information about the procedures involved. If you wish to read the research on which this study is based, please ask your study nurse or doctor.

Site Study Nurse Details

Principal Investigator (site) details:

Alternatively if you or your relatives have any questions about this study you may wish to contact one of the following organisations that are independent of the hospital at which you are being treated:

Prostate Cancer UK – 0800 074 8383 - http://prostatecanceruk.org

Macmillan Cancer Support - 0808 808 0121 – http://www.macmillan.org.uk

If you decide you would like to take part then please read and sign the consent form. You will be given a copy of this information sheet and the consent form to keep. A copy of the consent form will be filed in your patient notes, one will be filed with the study records and one may be sent to the Research Sponsor.

You can have more time to think this over if you are at all unsure.

Thank you for taking the time to read this information sheet and to consider this study.

Appendix 3 – Biological specimens

This will only be carried out if funding for storage and processing of the specimens is obtained.

Though not related to the primary or secondary outcomes of this study, UK participants will be consented to provide a urine, blood and semen sample after the consent and screen visit for storage and use in future biomarker studies. Samples will be stored in the Human Tissue Authority authorized UCL-Royal Free Hospital biobank, Research Tissue Bank approval number 11/WA/007. In addition, men will be consented for use of the prostate biopsy tissue in the biomarker discovery studies. No additional prostate biopsy samples will be taken for research. The biopsy tissue that is already taken for usual care is no longer required after usual care analysis. At this point we would store this tissue in the research biobank. These samples are optional and men do not need to provide these samples to be able to take part in the study.

The samples that would be collected includes:

Blood –5mls serum, 5mls plasma, 5mls whole blood,
Urine – 50mls urine
Semen – 1.5mls semen
