# Supplementary material for: A multicentre randomised controlled trial assessing whether MRI-targeted biopsy is non-inferior to standard transrectal ultrasound guided biopsy for the diagnosis of clinically significant prostate cancer in men without prior biopsy: a study protocol
Source: BMJ Open. 2017 Oct 12;7(10):e017863. doi: 10.1136/bmjopen-2017-017863 (PMC5706484; doi:10.1136/bmjopen-2017-017863)
Supplement: Reviewer comments [file bmjopen-2017-017863.reviewer_comments.pdf]

PEER REVIEW HISTORY

BMJ Open publishes all reviews undertaken for accepted manuscripts. Reviewers are asked to complete a checklist review form (**http://bmjopen.bmj.com/site/about/resources/checklist.pdf**) and are provided with free text boxes to elaborate on their assessment. These free text comments are reproduced below.

ARTICLE DETAILS

**TITLE (PROVISIONAL)**	A multi-centre randomised controlled trial assessing whether magnetic resonance imaging-targeted biopsy is non-inferior to standard trans-rectal ultrasound guided biopsy for the diagnosis of clinically significant prostate cancer in men without prior biopsy: a study protocol
**AUTHORS**	Kasivisvanathan, Veeru; Jichi, Fatima; Klotz, L.; Villers, Arnauld; Taneja, Samir; Punwani, Shonit; Freeman, Alex; Emberton, Mark; Moore, Caroline

This article was not externally reviewed at BMJ Open. Protocols that have been independently assessed prior to submission to BMJ Open are usually fast-tracked to publication on the grounds that further substantial changes will not be possible. This independent assessment will usually be external, independent review for both a substantial grant award from a non-commercial or government funder and ethics approval.
